# Primary Squamous Cell Carcinoma of the Cecum: A Case Report

**DOI:** 10.7759/cureus.54615

**Published:** 2024-02-21

**Authors:** Mahdi Albandar, Salwa Aljarayhi

**Affiliations:** 1 Department of Surgery, King Saud Medical City, Riyadh, SAU; 2 Department of Medicine, Al Faisal University, Riyadh, SAU

**Keywords:** perforation, colon, small bowel, cecum, primary, squamous cell carcinoma

## Abstract

Primary squamous cell carcinoma (SCC) of the colon is an exceptionally rare diagnosis. The etiology and pathogenesis of this entity remain unclear. It usually presents in patients as an emergency, typically with the tumor in the advanced stage. We report a case of SCC of the cecum presenting with perforation, initially diagnosed as SCC of unknown origin. The patient underwent a limited right hemicolectomy and end ileostomy outside our center. The patient was referred to us for further workup and possible adjuvant chemotherapy. She was assessed clinically and found to have had poor appetite and anorexia for a month, with an intermittent fever documented at 39 degrees. Thus, the patient was elected to get admitted for a septic workup and re-staging by CT scan and tumor biomarkers. CT showed a phlegmon and abscess formation at the right iliac fossa that was attached to surrounding structures, including the abdominal wall. Drain placement at the site of the phlegmon was attempted but failed due to bowel overlapping. Therefore, the patient was booked for surgical exploration and drainage, where all structures were resected en bloc. Histopathological examination revealed well-differentiated keratinized SCC with lymph node metastasis. The diagnosis of primary SCC of the cecum was confirmed after investigations to rule out primary sources were negative. Surgical resection remains the mainstay of management, with a possible role for chemotherapy and radiation therapy. The prognosis in these cases is usually poor. This warrants early diagnosis and management. Studies are needed to establish a management protocol for this entity.

## Introduction

Primary squamous cell carcinoma (SCC) of the colon is an extremely rare and intriguing diagnosis, with an incidence rate of 0.10-0.25 per 1,000 diagnosed colorectal carcinomas [1]. The etiology and pathogenesis of this entity remain unclear; however, it has been linked to inflammatory processes, infectious conditions, radiation exposure, and the development of squamous differentiation in pre-existing colonic adenomas [2]. Primary SCC of the colon often presents at advanced stages, mirroring the diagnostic complexity and urgency surrounding this condition. Herein, we report a case of primary SSC of the ascending colon and synchronous presence of SCC of the small bowel presenting as perforation at initial diagnosis and later presenting to our center with phlegmon formation following resection, which was raising suspicion of residual tumor.

## Case presentation

**Figure 1:**
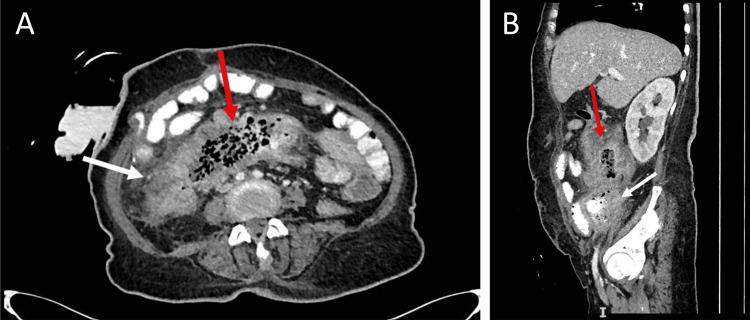
CT abdomen with oral contrast showing the abdominal collection, phelgmon, and fistulisations 1A: Cross-sectional CT image of the abdomen. 1B: Sagittal section CT image of the abdomen. Right iliac fossa phlegmon (white arrow); large mesenteric collection (red arrow) CT: computed tomography

A 59-year-old female is a known case of type 2 diabetes and hypertension. She first presented to another hospital with spontaneous perforation of the cecum, where she underwent a limited right-sided hemicolectomy and ileostomy creation. The reason why the initial managing team opted for a limited rather than a proper D2 right hemicolectomy is still unclear to us. Based on her histopathological features, she was diagnosed with cecal SCC. An extensive work-up to rule out a primary source was done. This included thorough CT scans of the head, neck, chest, and abdomen, as well as tumor marker tests for carcinoembryonic antigen, cancer antigen 19-9, and cancer antigen 125, all of which were negative. Additionally, negative results were observed in the upper gastrointestinal (GI) endoscopy, colonoscopy, thyroid scan, and breast scan, further ruling out potential sources. Immunohistochemistry of the resected specimen was done, which was positive for SCC markers. Thus, a diagnosis of unknown primary SCC was established initially. The patient was referred to us for further workup and possible adjuvant chemotherapy. Given the rarity of SCC in the colon, there was an initial assumption that the colonic mass might be metastatic in origin. Subsequently, multidisciplinary team discussions were conducted, in addition to family counseling, and the decision to initiate adjuvant chemotherapy was made. However, before starting her chemotherapy regimen, she presented at our hospital's emergency department complaining of decreased oral intake, abdominal pain, and diarrhea. She was febrile and dehydrated; otherwise, her physical exam was normal, with a soft, lax abdomen and no tenderness. The stoma looked healthy, with no signs of inflammation. She was elected to get admitted for a septic workup and re-staging based on a CT scan and tumor biomarkers. A CT scan showed a phlegmon and abscess formation at the right iliac fossa that was attached to surrounding structures, in addition to multiple intraabdominal collections and fistulisations (Figure 1A-1B). Drain placement at the site of the phlegmon was attempted but failed due to bowel overlapping. Therefore, the patient was booked for surgical exploration and drainage, where all structures were resected en bloc.

Intraoperatively, a well-capsulated deep fecal collection was seen at the root of the mesentery, forming phlegmon with multiple fistulas (entero-colic and entero-enteric) and attaching to the abdominal wall at the right iliac fossa area. Resection and anastomosis were done for the affected intestinal segments, including the hepatic flexure and part of the small intestine, in an en bloc resection manner, in addition to the affected part of the abdominal wall. Due to the involvement of the ileostomy part within the phlegmon, it was all taken down, and a new ileostomy was fashioned again. Drains were placed in the pelvis and the previous collection cavity.

**Figure 2:**
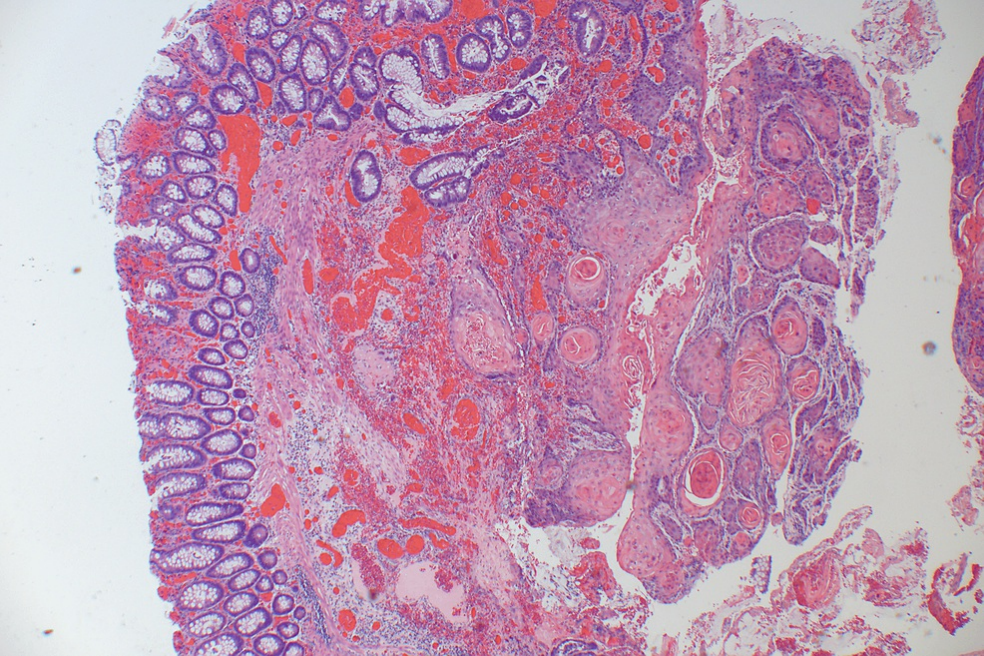
Hematoxylin and eosin (H&E) staining of the resected right iliac fossa mass A micrograph at 4x magnification shows well-differentiated squamous cells with keratin pearls

The histopathology report of specimens received from her initial managing hospital reviewed at our hospital suggested a well-differentiated keratinizing SCC of the ileocecal region that could be metastatic with few glandular components (Figure 2), with no areas of adenocarcinoma. One lymph node among the three lymph nodes resected showed tumor involvement, suggesting tumor metastasis. A vascular invasion was identified. Specimens obtained during the patient's second surgical exploration at our hospital, including the colonic hepatic flexure and a mass in the small bowel, revealed consistent findings indicating the synchronous presence of primary SCC in the resected portion of the right colon and small bowel. Five out of eleven resected lymph nodes were positive for metastatic keratinizing SCC. The patient underwent an extensive assessment to determine a potential primary source of malignancy; however, all evaluations were negative. The patient lacked any skin lesions or ulcers that would point to the skin as the primary source.

The postoperative course was uneventful initially; however, later on, she developed frequent vomiting and could not tolerate it well orally. The patient experienced postoperative complications, including wound infection and collection, both effectively addressed through the administration of broad-spectrum antibiotics and drainage. Furthermore, she developed pneumonia and pleural effusion, and thus chest physiotherapy was initiated. The patient was re-staged and found to have evidence of peritoneal deposits, as shown by a CT scan. Patient data and clinical information were discussed in detail during tumor board discussions, and patient counseling was conducted for further management plans. The patient will be started on adjuvant chemotherapy at the following scheduled visit.

## Discussion

Primary SSC of the GI tract is a rare finding in the colon and rectum; however, it is a very common malignancy in the esophagus and anus. The first case of pure SCC in the colon was reported back in 1919 by Schmidtmann [3]. Since then, around 150 cases of colorectal SCC have been reported in the literature, with the majority of these cases describing SCC in the rectum. The right colon is the second-most common site for colorectal SCC [1]. Approximately only 64 papers describing colonic SSC are present in the English literature [4-5].

SSC of the colon typically manifests, on average, in the fifth decade of life, displaying a higher occurrence among males and a greater predilection for the cecum and the right colon [6-8]. Most cases of colon SCC are diagnosed in advanced stages. Patients typically present with symptoms similar to those of other colorectal neoplasms like adenocarcinomas, which include abdominal pain, changes in bowel habits, rectal bleeding, and weight loss. A minority of cases, however, manifest as acute surgical emergencies, such as obstruction or perforation (as in our presented case) [9]. These emergencies are more common with SCC of the colon due to the more aggressive nature of this tumor. Only two cases of cecal perforation in primary cecal SCC have been found in the literature review, and our case is the third [2,10].

The pathogenesis of primary SCC of the colon remains unclear to this day; however, several hypotheses have been suggested, including chronic GI inflammatory processes such as those seen with ulcerative colitis and infectious etiologies (e.g., schistosomiasis and *Entamoeba histolytica*). These two entities are proposed to cause squamous metaplasia, which then progresses to carcinoma [11-12]. A history of radiation therapy or previous surgery has also been noted in patients with SCC of the colon [13]. An alternative hypothesis suggests that SSC could arise as a result of squamous differentiation of pluripotent stem cells or of pre-existing colonic adenomas or adenocarcinomas [12,14-15].

Considering the uncommon occurrence of this entity, certain criteria must be fulfilled before making the diagnosis of primary/pure SCC of the colon. Williams et al. (1979) proposed criteria for the diagnosis of primary SCC in the colon, which include: (1) absence of SCC in any other organ that could cause direct spread/invasion into the bowel or provide a source for colonic metastasis. Possible primary sites include skin, ovary, cervix, bladder, lungs, and esophagus; (2) absence of any squamous-lined fistula tract involvement in the affected bowel segment, as this can be the source of the squamous carcinoma; (3) in the case of rectal SCC, proximal extension of anal SCC should be excluded; and lastly, (4) confirmation of SCC through histological analysis [16]. These criteria were all fulfilled in our case.

Management guidelines have not yet been established for this condition due to its rarity. The mainstay of managing SCC of the colon is agreed to be surgery by most authors. The benefit of treatment with adjuvant chemotherapy and radiation therapy has not been supported by evidence yet; however, several cases have demonstrated tumor response and better overall patient outcomes [17-18]. Chemotherapeutic agents mentioned in the literature included 5-fluorouracil, mitomycin C, or cisplatin [11]. Pure colonic SSC has a worse prognosis compared to adenocarcinoma, with an overall five-year survival rate of 30-35% after surgery and a recurrence rate of approximately 80% in a three-year period [2,19].

Surgical site infection is the most common postoperative complication after colectomies, causing pain and suffering for patients. In addition, this complication has been associated with negative economic impact, increased morbidity, extended postoperative hospital stay, readmission, sepsis, and death [20].

Referring the patient to the necessary specialty is a safe and effective approach to preventing avoidable complications. Making a timely decision for surgery following the completion of the workup is a crucial step. The persistence of an unresolved phlegmon raises clinical suspicion of malignancy, warranting consideration for surgical exploration to exclude the possibility of recurrent or residual malignancy.

## Conclusions

SCC of the colon is a rare entity with unclear etiology and pathogenesis. This rare tumor is aggressive in nature and usually presents in late stages. The prognosis of patients with colonic SCC is worse than that of adenocarcinoma, with a five-year survival rate of only 30%. Management guidelines have not yet been established for this disease; however, surgery remains the mainstay of management. Further studies are needed to understand its pathogenesis, and clinical trials ought to be conducted on such cases to facilitate the establishment of a clear management protocol.
